# Supplementary material for: Excitatory Neuron-Derived Interleukin-34 Controls Cortical Developmental Microglia Function
Source: bioRxiv. 2025 May 7:2024.05.10.589920. Originally published 2024 May 10. Preprint. [Version 2] doi: 10.1101/2024.05.10.589920 (PMC11100801; doi:10.1101/2024.05.10.589920)
Supplement: Supplement 1 [file NIHPP2024.05.10.589920v2-supplement-1.pdf]

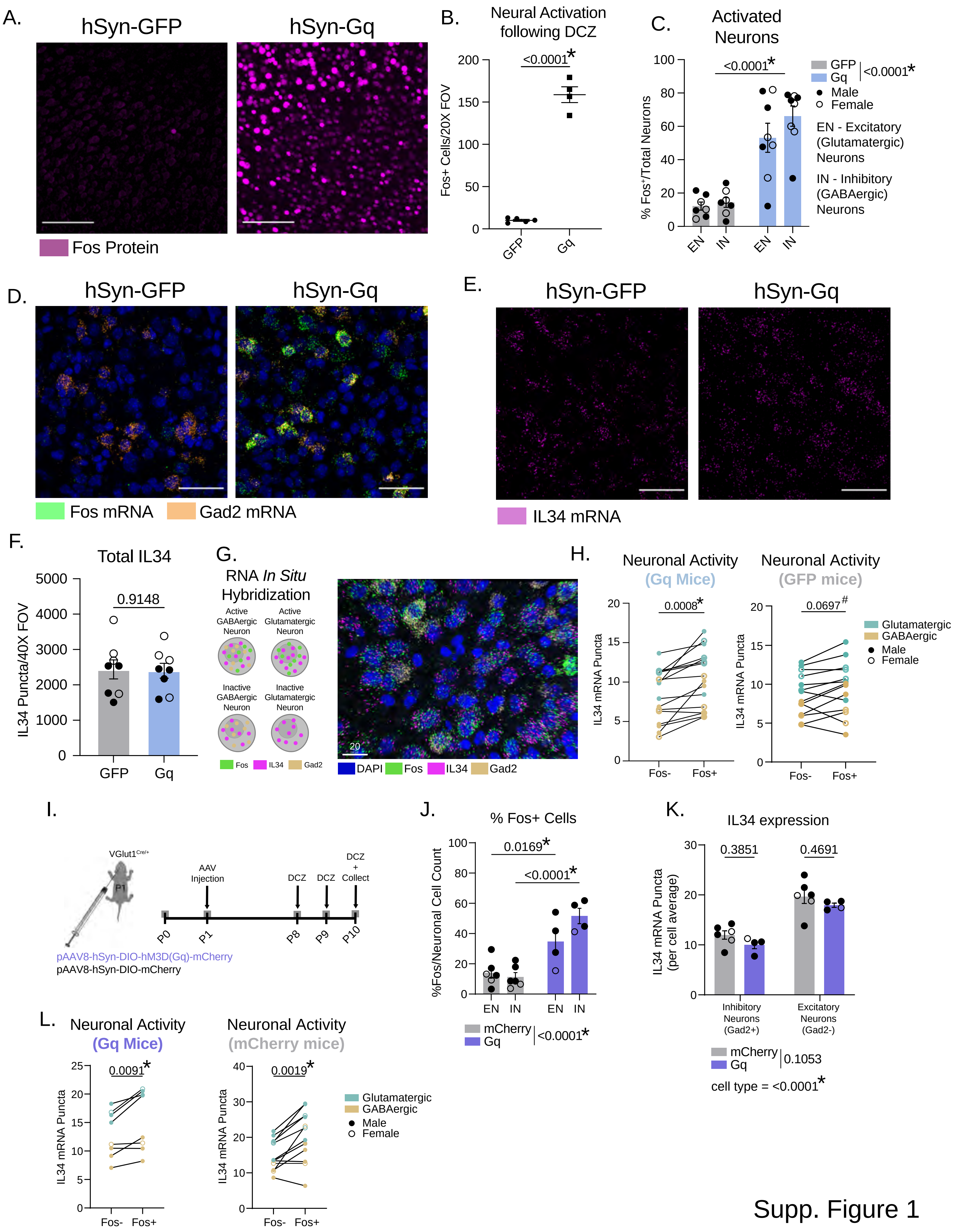

Supp. Figure 1

Supplemental Figure 1. IL34 expression is increased in Fos+ neurons. Related to Figure 1.
(A-B) Representative images and quantification of Fos protein in GFP-control mice and Gq- DREADD mice. (n = 4-5 mice/virus, unpaired t-test). Scale = 100uM
(C-D) Quantification and representative images of Fos mRNA in excitatory and inhibitory neurons in GFP and Gq mice. (n=3-4 mice/sex/virus, one-way ANOVA, main effect of virus in legend). Scale = 50uM
(E-F) Total IL34 puncta per 40X field of view in GFP control and Gq mice. (n=3-4 mice/sex/virus, unpaired t-test). Scale = 50uM
(G) Schematic outlining the different cell types identified by expression of IL34, Gad2, and Fos using RNA-FISH and representative image of RNA-FISH stain showing IL34 levels in Fos+ (active) and Fos- (inactive) neurons.
(H) Quantification of IL34 mRNA puncta in Fos- and Fos+ cells in mice that received the Gq or GFP virus. (n = 4 mice/sex, data shown are a per animal average of the expression level of all neurons of that type (excitatory vs. inhibitory, and active vs. inactive, total of 5,722 cells analyzed), paired t-test).
(I) Schematic of excitatory neuron-specific chemogenetic activation experiments.
(J) Quantification of % Fos+ cells in excitatory and inhibitory neurons in mCherry and Gq mice. (n=1-4 mice/sex/virus, data shown are an average of 3 images taken from 3 sections of each mouse, two-way ANOVA, Sidak's post-hoc test, main effect of virus shown in legend).
(K) Quantification of average IL34 expression in excitatory and inhibitory neurons in mCherry and Gq mice. (n=1-4 mice/sex/virus, data shown are an average of 3 images taken from 3 sections of each mouse, two-way ANOVA, Sidak's post-hoc test, main effect of virus in legend).
(L) Quantification of IL34 mRNA puncta in Fos- and Fos+ cells in mice that received the Gq or control virus. (n=1-4 mice/sex/virus, data shown are a per animal average of the expression level of all neurons of that type (excitatory vs. inhibitory, and active vs. inactive, total of 2,566 cells analyzed), paired t-test).

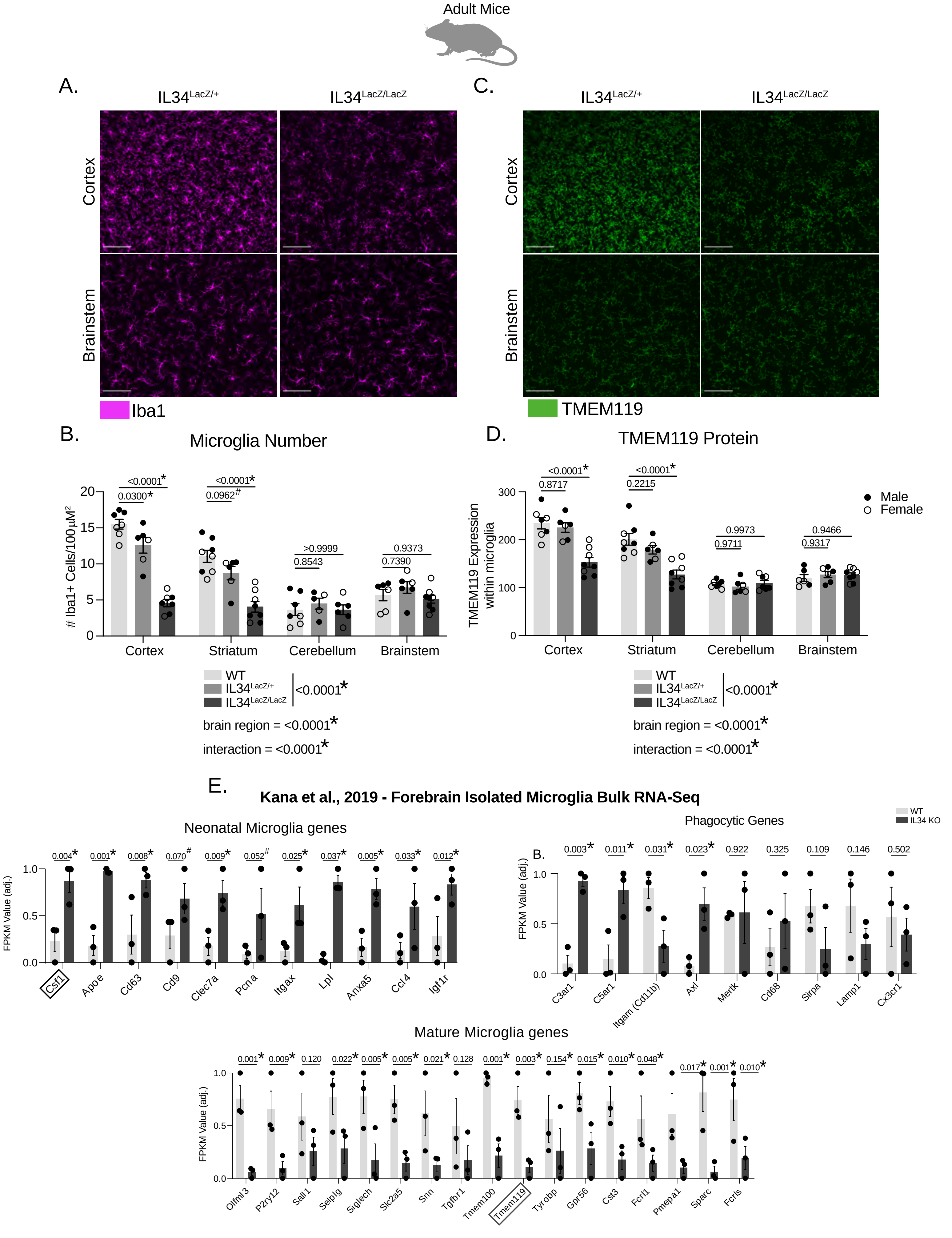

Supp. Figure 2

Supplemental Figure 2. IL34 KO impacts microglia numbers and TMEM119 expression in adult cortex and striatum, but not cerebellum or brainstem. Related to Figure 2.
(A and C) Representative images of Iba1 and TMEM119 stain in WT, IL34$^{LacZ/+}$ and IL34$^{LacZ/LacZ}$ mice from both the cortex and brainstem. Scale bar = 100µM.
(B and D) Quantification of microglia number and TMEM119 mean gray value in the cortex, striatum, cerebellum, and brainstem from WT, IL34$^{LacZ/+}$ and IL34$^{LacZ/LacZ}$ mice. (n = 2-4 mice/sex/genotype, two-way ANOVA, Sidak's post-hoc test, main effect of genotype and brain region and interaction in legend).
(E) Scaled gene expression (FPKM) of embryonic/neonatal and adult microglia markers and candidate phagocytic genes from forebrain microglia from WT and IL34$^{LacZ/LacZ}$ (IL34 KO) male mice. P values shown are from multiple t-tests function in Graphpad Prism. List of marker genes compiled from sequencing done in Bennett et al., 2016, Matcovitch-Natan 2014, and Li et al., 2019. Data from this figure was originally generated and published in Kana et al., 2019 and is accessible at GSE133362.

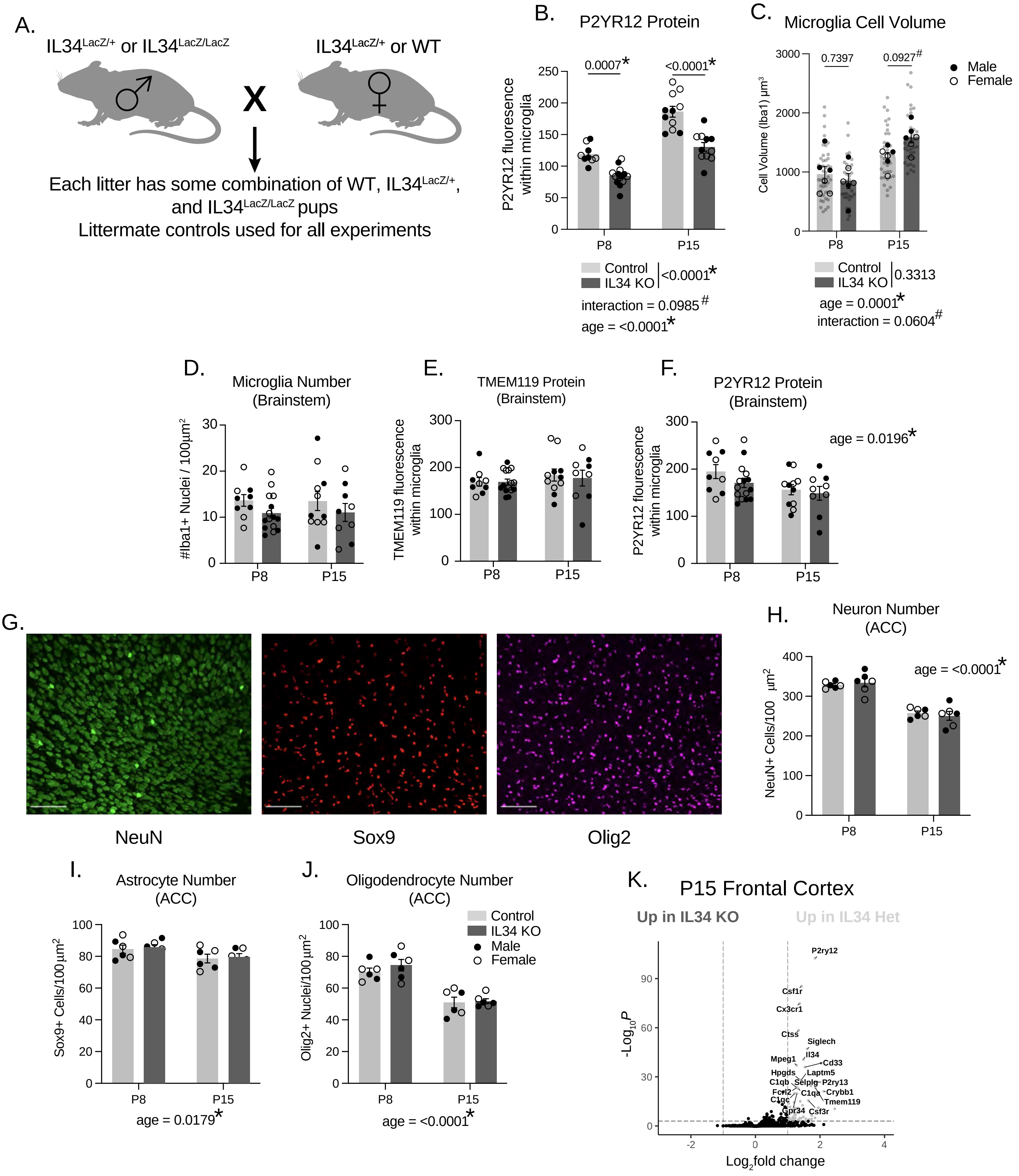

Supp. Figure 3

Supplemental Figure 3. IL34 KO specifically impacts microglia in the forebrain. Related to Figure 2.
(A) Representative schematic of $IL34^{LacZ/LacZ}$ breeding scheme.
(B) Quantification of P2YR12 mean gray value in the ACC of P8 and P15 IL34 KO and Control mice (n = 5-8 mice/sex/age/genotype, two-way ANOVA age x genotype, Sidak's post-hoc test, main effect of genotype and age and interaction term in legend).
(C) Quantification of microglia Iba1 cell volume from IMARIS 3D reconstructions. (n = 3 mice/sex/age/genotype, 4-6 cells analyzed per mouse, individual microglia represented by gray circles, animal averages represented by black dots, two-way ANOVA age x genotype, Sidak's post-hoc test, main effect of genotype and age and interaction term in legend).
(D-F) Quantification of microglia cell number, TMEM119 expression, and P2YR12 expression from the brainstem of P8 and P15 control and IL34 KO mice. (n = 5-8 mice/sex/age/genotype, two-way ANOVA age x genotype).
(G) Representative images of NeuN, Sox9, Olig2 triple stain for quantification of neuron, astrocyte, and oligodendrocyte numbers/density. Scale bar = 100µM.
(H-J) Quantification of Neuron, Astrocyte, and Oligodendrocyte numbers in ACC of P8 and P15 control and IL34 KO mice. (n=3 mice/sex/age/genotype, data shown are an average of 3 images taken from 3 sections of each mouse, two-way ANOVA age x genotype, main effect of age and interaction term in legend where significant).
(K) Bulk RNASequencing was performed on a separate cohort of $IL34^{LacZ/+}$ and $IL34^{LacZ/LacZ}$ mice at postnatal day 15. (n=2 mice/sex/genotype, genes shown as significant passed a threshold of padj < 0.001 and LogFC > 1).

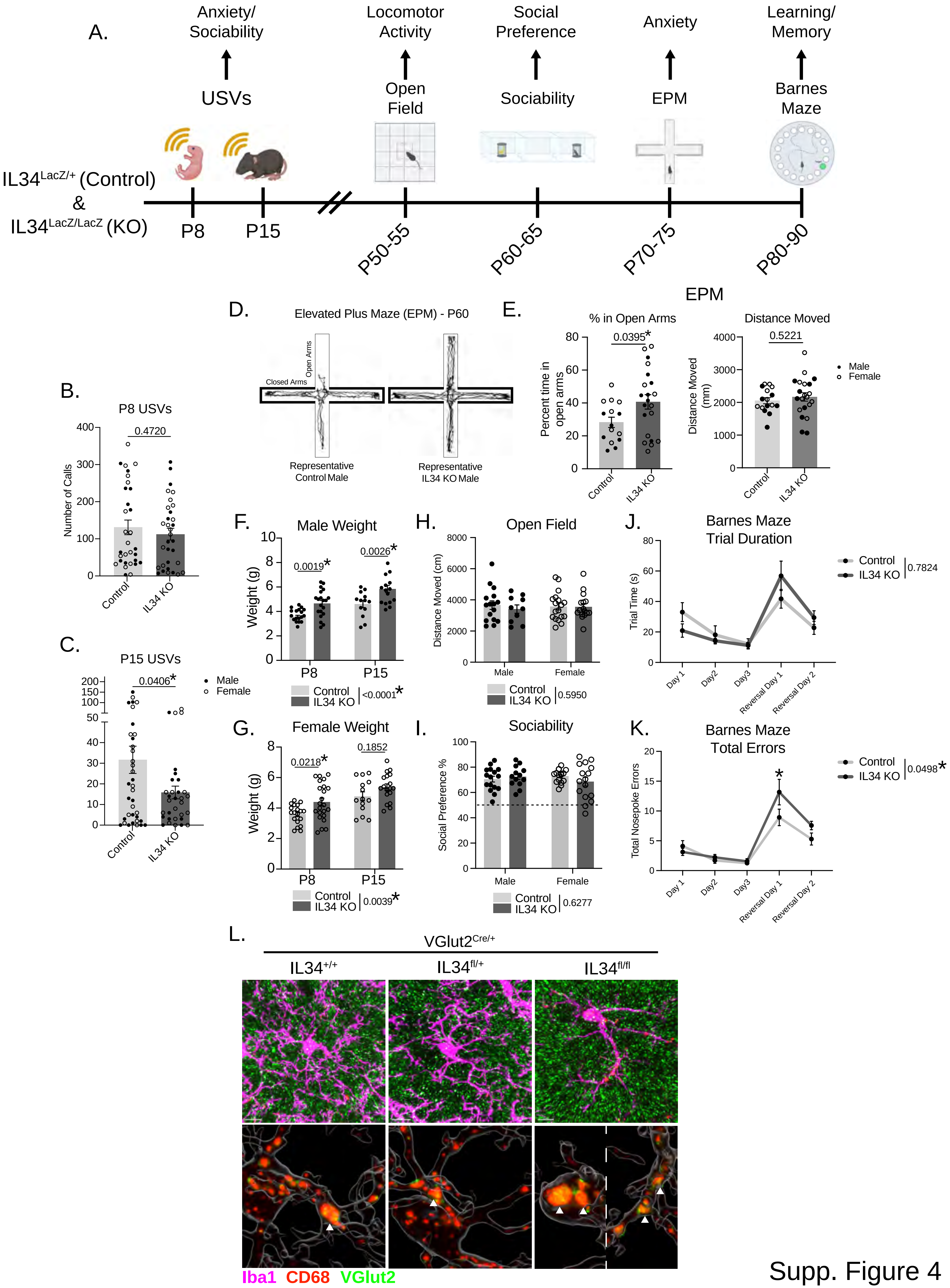

Supp. Figure 4

Supplemental Figure 4. IL34 KO impacts pup weight, ultrasonic vocalizations, and anxiety in adulthood. Related to Figure 2.
(A) Experimental timeline for IL34 KO behavioral phenotyping in development and adulthood.
(B-C) Quantification of the total number of vocalizations made over the three-minute isolation period at P8 and P15 in IL34 KO and control mice. (n = 16-18 mice/sex/genotype, unpaired t tests).
(D) Representative movement traces in the Elevated Plus Maze test in adult control and IL34 KO mice.
(E) Quantification of percent time spent in open arms of the EPM and total distance moved over the 5-minute test (time spent in open arms / total test time * 100). (n = 6-12 mice/sex/genotype, unpaired t test).
(F-G) Quantification of Male and Female pup weights from control and IL34 KO mice at P8 and P15. (n = 16-18 mice/sex/genotype, two-way ANOVA age x genotype, main effect of genotype in legend).
(H) Quantification of total distance moved over a ten-minute period in the open field. (n = 10-16 mice/sex/genotype, two-way ANOVA sex x genotype, main effect of genotype in legend).
(I) Quantification of social behavior from the three-chamber sociability assay. (n = 10-16 mice/sex/genotype, two-way ANOVA sex x genotype, main effect of genotype in legend).
(J-K) Quantification of performance (trial time and errors) of male control and IL34 KO mice in the barnes maze across three training days and two reversal learning days. (n=8 male mice/genotype, two-way ANOVA day x genotype, main effect of genotype in legend, significance of errors on reversal day 1 from Sidak's post-hoc test).
(L) Raw IMARIS representative images of VGlut2 synaptic material engulfed in CD68+ lysosomes within microglia in the ACC of $VGlut2^{Cre}$ $IL34^{+/+}$, $IL34^{fl/+}$, and $IL34^{fl/fl}$ mice.

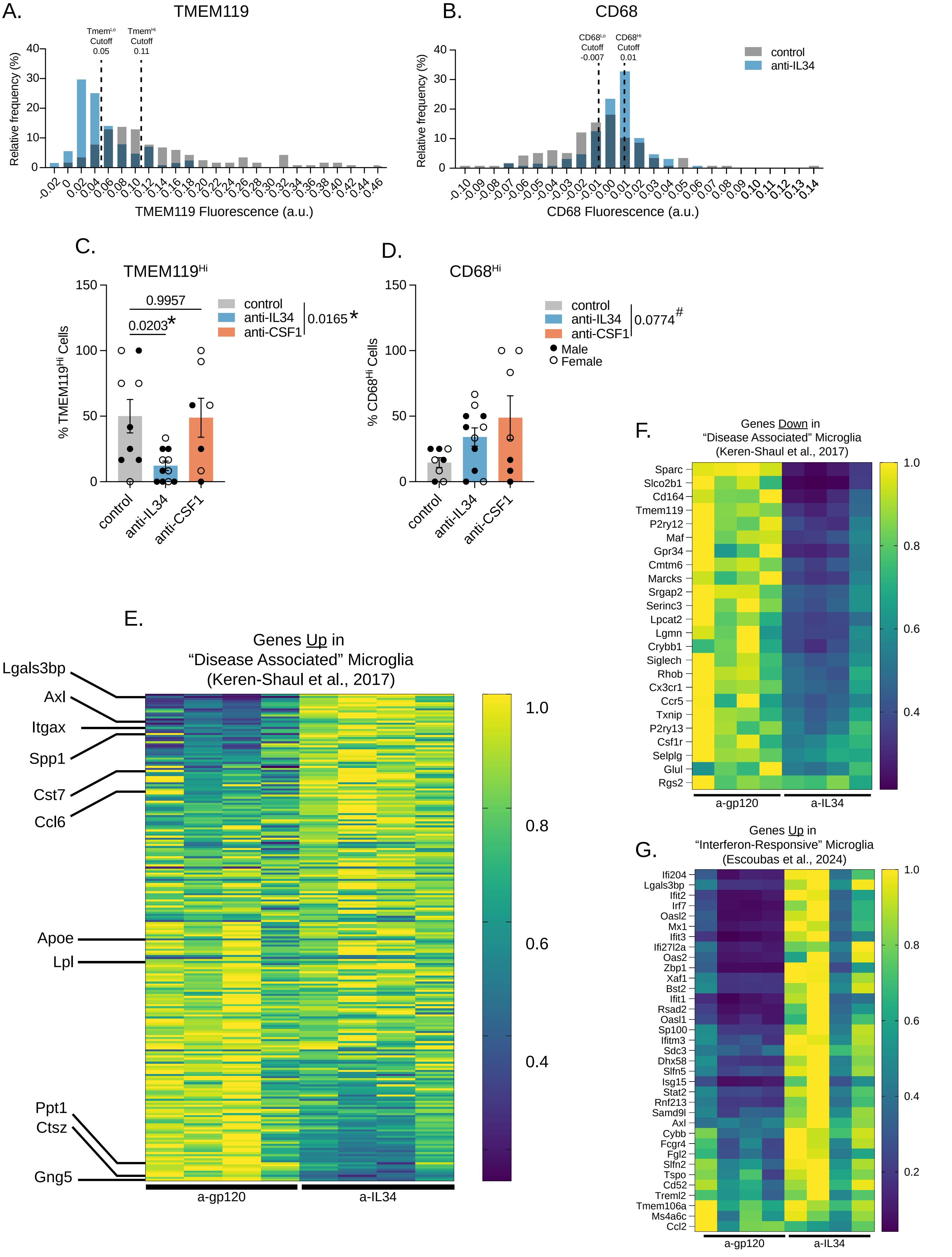

Supp. Figure 5

Supplemental Figure 5. anti-IL34 microglia share some transcriptional similarities with disease-associated microglia and interferon-responsive microglia. Related to Figure 5.
(A-B) Histograms of relative fluorescence of TMEM119 and CD68 stain within individual microglia.
(C-D) Quantification of $TMEM119^{Hi}$ and $CD68^{Hi}$ microglia from control, anti-IL34, and anti-CSF1 mice. (n = 3-6 mice/sex/antibody, data shown are a percentage of 12 cells measured per animal across 3 images, one-way ANOVA, Sidak's post-hoc test, main effect of antibody in legend).
(E-G) Heatmaps of normalized TPM values between a-gp120 (control) and a-IL34 isolated microglia including all upregulated or downregulated genes from the "disease-associated" microglia cluster from Keren-Shaul et al., 2017, and all upregulated genes from the "interferon-responsive" microglia cluster from Escoubas et al., 2024.

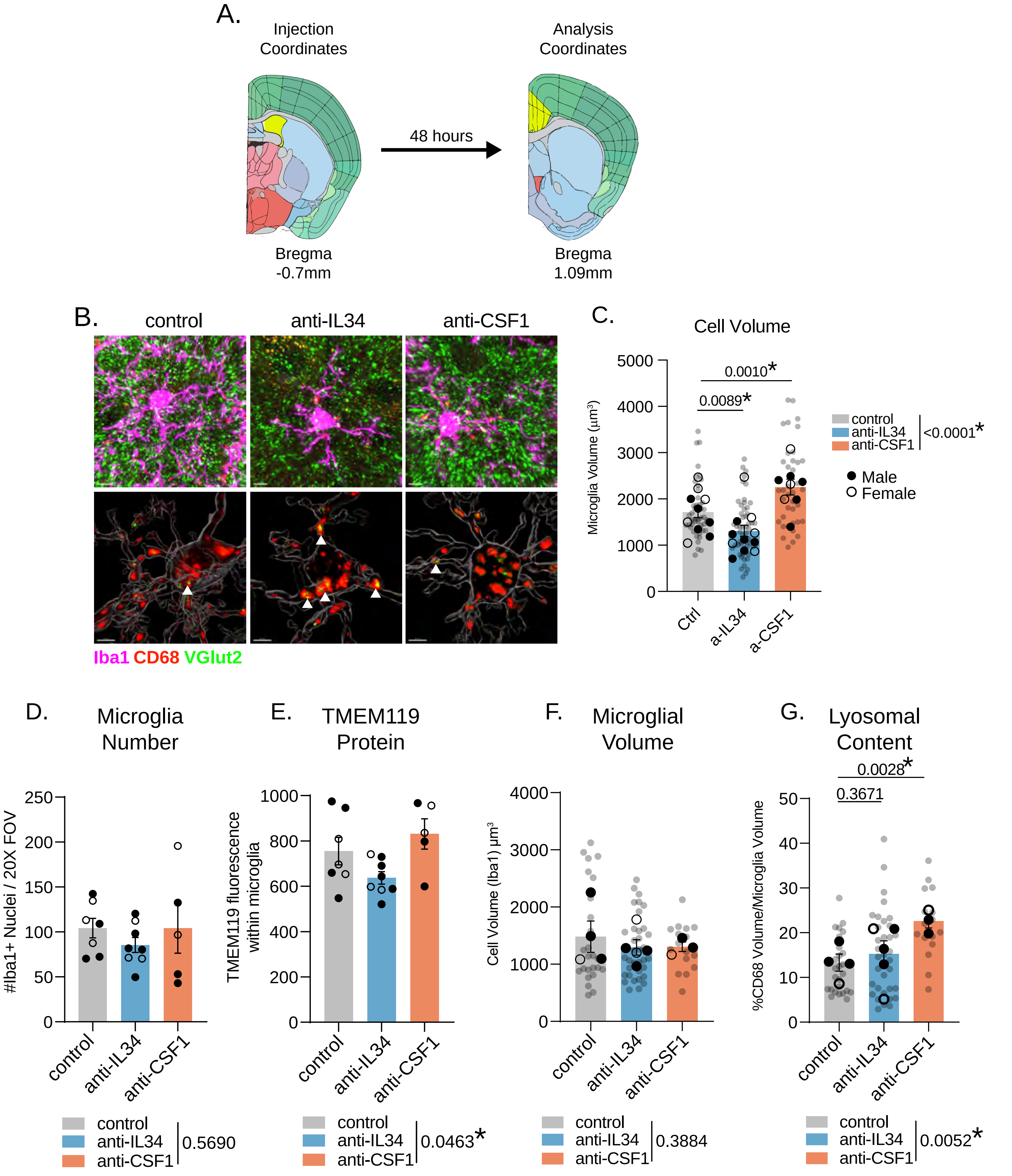

Supp. Figure 6

Supplemental Figure 6. anti-IL34 does not impact cell number, but reduces TMEM119 expression and increases synaptic engulfment in the hippocampus. Related to Figure 6.
(A) Representative atlas coordinates for lateral ventricle injection and ACC analysis. Images courtesy of Allen Brain Atlas.
(B) Raw fluorescent representative images of microglia from control, anti-IL34, and anti-CSF1 mice. (C) Quantification of microglial cell volume. (n = 3-5 mice/sex/antibody, 4-6 cells analyzed per mouse, 141 cells total, individual microglia represented by gray circles, animal averages represented by black dots, nested one-way ANOVA, Sidak's post-hoc test, main effect of antibody in legend).
(D-E) Quantification of microglia number and TMEM119 protein in the hippocampus. (n = 2-5 mice/sex/antibody, one-way ANOVA, main effect of antibody in legend).
(F-G) Quantification of microglia volume and lysosomal content in hippocampal microglia from control, anti-IL34, and anti-CSF1 mice. (n = 1-3 mice/sex/antibody, 4-6 cells analyzed per mouse, individual microglia represented by gray circles, animal averages represented by black dots, nested one-way ANOVA, Sidak's post-hoc test, main effect of antibody in legend).

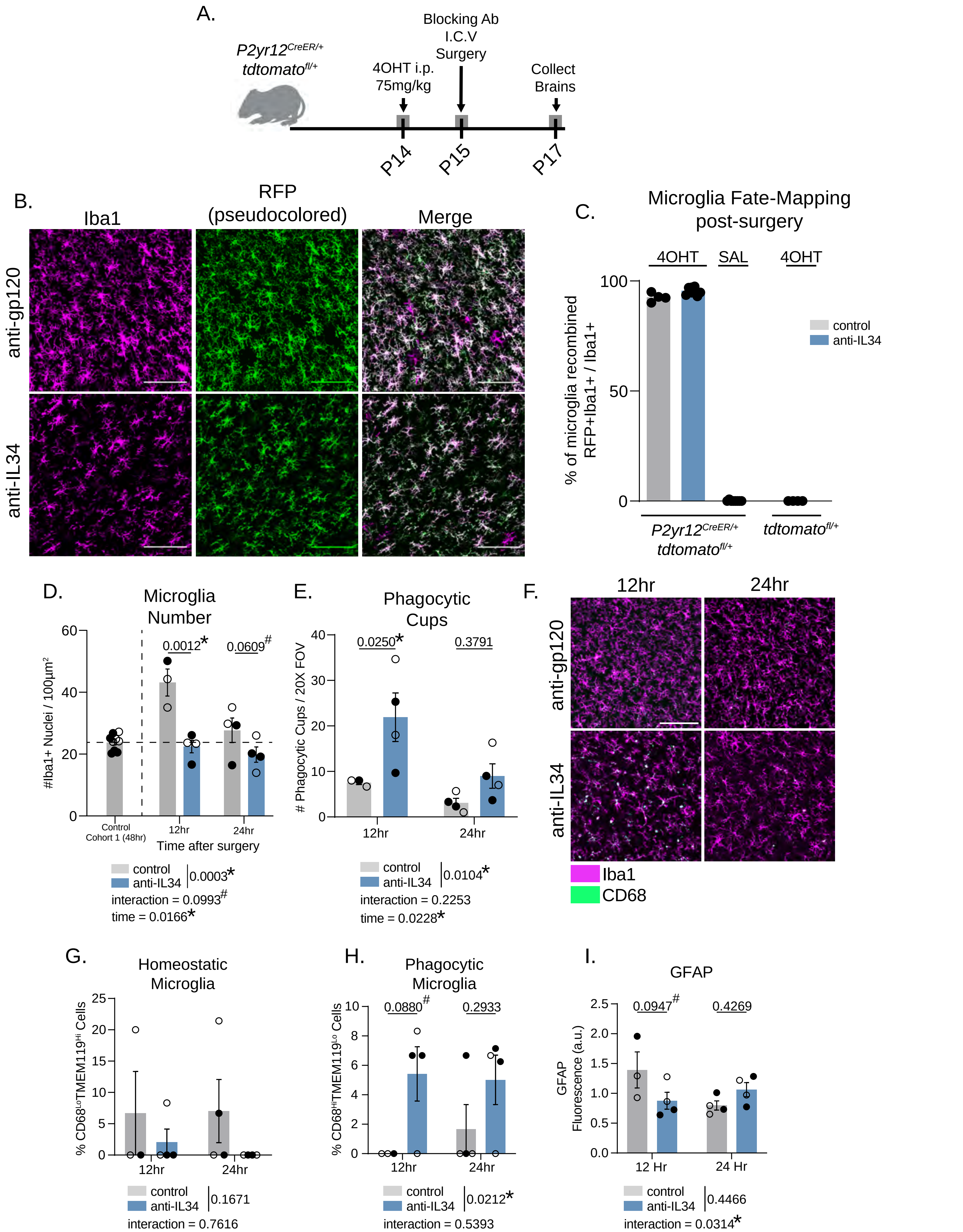

Supp. Figure 7

J.

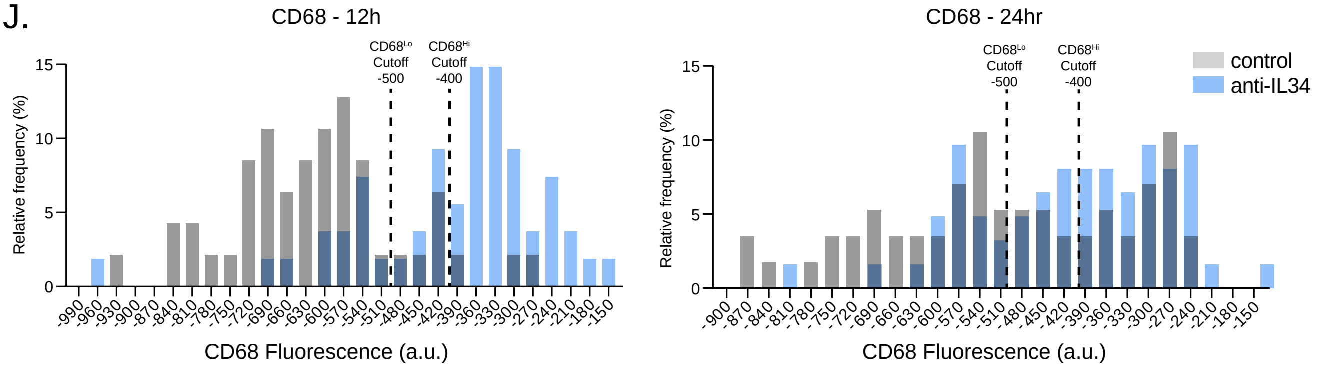

K.

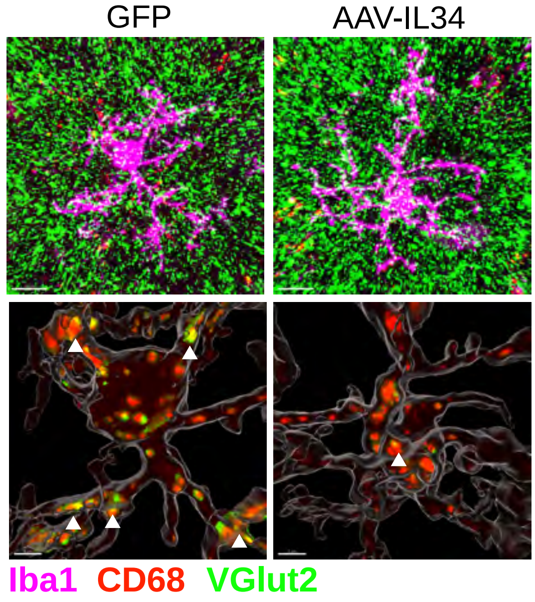

Supplemental Figure 7. anti-IL34 treatment does not cause mass microglial death and repopulation. Related to Figure 4, 5, and 6.

(A) Experimental schematic of 4-hydroxytamoxifen injection, blocking antibody surgery, and tissue collection.

(B) Representative images of Iba1 and RFP staining in the ACC of mice treated with either the control or anti-IL34 blocking antibody. Scale = 100uM

(C) Quantification of the percentage of microglia (total Iba1+ cells) that were also RFP+ following blocking antibody administration.

(D) Quantification of microglia number in mice treated with a control or anti-IL34 blocking antibody 12- and 24-hours following surgery. Microglia number from controls from the original cohort collected 48 hours post-surgery is also shown. (n = 3-4 mice/timepoint/antibody, two-way ANOVA, Sidak's post-hoc test, main effect of antibody and timepoint and interaction effect in legend).

(E) Quantification of the number of phagocytic cups within 20X field-of-view. (n = 3-4 mice/timepoint/antibody, two-way ANOVA, Sidak's post-hoc test, main effect of antibody and timepoint and interaction effect in legend).

(F) Representative images of Iba1 and CD68 staining in mice treated with a control or anti-IL34 blocking antibody 12- and 24-hours following surgery.

(G-H) Quantification of CD68^{Lo}TMEM119^{Hi} homeostatic microglia and CD68^{Hi}TMEM119^{Lo} phagocytic microglia in all four groups. (n = 3-4 mice/timepoint/antibody, data shown are a percentage of 12 cells measured per animal across 3 images, two-way ANOVA, Sidak's post-hoc test, main effect of antibody and timepoint and interaction effect in legend).

(I) Quantification of GFAP mean gray value in all four groups. (n = 3-4 mice/timepoint/antibody, two-way ANOVA, Sidak's post-hoc test, main effect of antibody and timepoint and interaction effect in legend).

(J) Histograms of relative fluorescence of CD68 stain within individual microglia at both timepoints.

(K) Raw IMARIS representative images of VGlut2 synaptic material engulfed in CD68+ lysosomes within microglia in control GFP and AAV-IL34 mice.
